# Space and space-time distributions of dengue in a hyper-endemic urban space: the case of Girardot, Colombia

**DOI:** 10.1186/s12879-017-2610-7

**Published:** 2017-07-24

**Authors:** Mauricio Fuentes-Vallejo

**Affiliations:** 1 Fundación Santa Fe de Bogotá, Bogotá, Colombia; 2 Laboratory of Social Dynamics and Spatial Reconstruction (LADYSS), Paris, France

**Keywords:** Spatial analysis, Urban space, Dengue, Colombia

## Abstract

**Background:**

Dengue is a widely spread vector-borne disease. Dengue cases in the Americas have increased over the last few decades, affecting various urban spaces throughout these continents, including the tourism-oriented city of Girardot, Colombia. Interactions among mosquitoes, pathogens and humans have recently been examined using different temporal and spatial scales in attempts to determine the roles that social and ecological systems play in dengue transmission. The current work characterizes the spatial and temporal behaviours of dengue in Girardot and discusses the potential territorial dynamics related to the distribution of this disease.

**Methods:**

Based on officially reported dengue cases (2012–2015) corresponding to epidemic (2013) and inter-epidemic years (2012, 2014, 2015), space (Getis-Ord index) and space-time (Kulldorff’s scan statistics) analyses were performed.

**Results:**

Geocoded dengue cases (*n* = 2027) were slightly overrepresented by men (52.1%). As expected, the cases were concentrated in the 0- to 15-year-old age group according to the actual trends of Colombia. The incidence rates of dengue during the rainy and dry seasons as well as those for individual years (2012, 2013 and 2014) were significant using the global Getis-Ord index. Local clusters shifted across seasons and years; nevertheless, the incidence rates clustered towards the southwest region of the city under different residential conditions. Space-time clusters shifted from the northeast to the southwest of the city (2012–2014). These clusters represented only 4.25% of the total cases over the same period (*n* = 1623). A general trend was observed, in which dengue cases increased during the dry seasons, especially between December and February.

**Conclusions:**

Despite study limitations related to official dengue records and available fine-scale demographic information, the spatial analysis results were promising from a geography of health perspective. Dengue did not show linear association with poverty or with vulnerable peripheral spaces in intra-urban settings, supporting the idea that the pathogenic complex of dengue is driven by different factors. A coordinated collaboration of epidemiological, public health and social science expertise is needed to assess the effect of “place” from a relational perspective in which geography has an important role to play.

**Electronic supplementary material:**

The online version of this article (doi:10.1186/s12879-017-2610-7) contains supplementary material, which is available to authorized users.

## Background

Dengue is a vector-borne disease that is widely spread. A recent estimate [1] indicated that 390 million dengue infections occur every year (95% CIs = 284–528 million), of which 96 million (95% CIs = 67–136 million) people manifest clinically severe forms of the disease. It is also predicted that dengue will be ubiquitous throughout the tropics, with specific local risk variations influenced by rainfall, temperature and the degree of urbanization. Although specific-type immunity follows dengue virus infection, an uneven spatial and temporal circulation exists regarding the four virus serotypes of dengue, resulting in endemic and epidemic transmission. According to the clinical manifestations of the disease, dengue is classified as either non-severe (with or without warning signs) or severe, which includes the life-threating manifestations of the disease [2].

The Americas have reported an increase in cases over the past 30 years and local transmission has been reported over the entire region, excluding Canada, Uruguay, and Chile [3, 4]. In 2013, more than 2 million dengue cases were reported in the Americas (of which 32,270 cases were severe), resulting in an incidence of 404.35 per 100,000 people and 1175 deaths (case fatality rate = 0.05%) [4]. The dengue mosquito vectors *Aedes albopictus* and *Aedes aegypti* are both present in the Americas, although the latter predominates, representing a major public health concern because it is also the vector associated with recent and rapidly spreading diseases such as chikungunya [5] and Zika [6]. During the 1950s and part of the 1960s, great efforts were undertaken to eradicate the vector *Aedes aegypti* from the Americas as part of the yellow fever eradication initiatives [7]. The weakening of surveillance systems and vector control programmes led to the re-infestation of *Aedes aegypti* over time, causing periodic outbreaks that now occur cyclically every three to 5 years [8]. Dengue is characterized as an emerging and re-emerging disease, and it has become a major public health problem because of its disease burden and the economic impact it has on the region [8–10].

*Aedes aegypti* is widespread in Colombia at altitudes less than 1800 m above sea level (MASL), and dengue is endemic throughout most of the country. Unlike other countries of the region, Colombia reports cases throughout the year, with increases during the rainy seasons [4]. It is estimated that more than half of the country’s population (approximately 24 of the total 46 million people) live in areas environmentally susceptible to dengue virus transmission [8]. According to Padilla [8], the number of cases and municipalities reporting dengue has increased over recent decades, whereas 66% of the country’s municipalities (*n* = 1112) reported cases in 2010. Twelve dengue epidemics were registered in Colombia between 1971 and 2010, totalling more than 1 million cases of dengue (annual average of 30,928 cases).

### Dengue in urban spaces

The theoretical framework of mosquito-transmitted diseases has evolved over the last few decades as a product of the permanent assessment of the new interactions between vectors and human biology. One issue that has accompanied the evolution of these theoretical models concerns the spatial and temporal dynamics of the transmission now considered heterogeneous (i.e., the interactions among mosquitoes, pathogens and human beings can predict different results). This issue has led to the analysis of transmission at different temporal and spatial scales and the roles of social and ecological systems; in other words, “Emerging theory focuses attention on the ecological and social context for mosquito blood feeding, the movement of both hosts and mosquitoes, and the relevant spatial scales for measuring transmission and for modeling dynamics and control” [11].

Nevertheless, different studies have shown opposite results regarding the possible relationships between the incidence of dengue and the presence of vector mosquitoes, socioeconomic conditions, urban morphology, and the knowledge, attitudes and practices of a population. These relationships remain to be established [12–16]. Vector control strategies have also been assessed, revealing that a unique effective and generalized prevention strategy that can sufficiently address dengue transmission in urban settings does not exist [17]. Hence, the spatial dimension has been recognized as essential to better understand the transmission of diseases such as dengue [11]. Geography itself has played a role in this debate via the classical works of Maximilien Sorre [18] and, more recently, with analyses of dengue discussing how particular configurations of each urban space influence the spatial pattern of the disease [7, 15, 16, 19, 20].

Based on officially reported dengue cases (2012–2015) corresponding to epidemic (2013) and inter-epidemic years (2012, 2014, 2015), the current work sought to characterize the spatial and temporal behaviours of dengue in Girardot, Colombia. Moreover, it discusses the potential territorial dynamics related to the distribution of this disease.

### Study area: Girardot in a regional setting

Girardot is located 134 km southwest of Bogotá (2 h’ drive), 289 MASL. Girardot belongs to the Department of Cundinamarca, but it shares natural (the Bogotá and Magdalena Rivers) and administrative limits with the Department of Tolima. Girardot is the most important city in its province; it has a population of 105,085 inhabitants, of whom 96.6% are concentrated in a 20-km^2^ urban area [21, 22]. Girardot has undergone a conurbation process with its neighbouring municipalities Ricaurte and Flandes (Department of Tolima). This process has engendered urban growth, which influences functional changes from rural to urban structures with recreational, commerce and service functions [23]. Characterized by a bimodal rain regime, the two rainy seasons occur from March to May and from October to November. The mean precipitation is 1220 mm, with relative humidity of 66.4% and a mean temperature of 33.3 °C.

Habituated to the constant population flux, the city provides services such as hotels and holiday centres, and it has evolved as an attractive municipality for the “second residences” of inhabitants of other municipalities (primarily Bogotá) for recreational purposes. As a result, the urban area of Girardot includes 5000 beds in more than 40 hotels and 55 closed residential areas (“condominios” and “conjuntos residenciales”), primarily used as secondary or vacation residences [24]. At the periphery of the urban area, extensive vacation/residential complexes are common, including El Peñon, which has approximately 900 houses. The city also provides administrative services to the province of Alto Magdalena as well as the Department of Cundinamarca, grouping all types of environmental, tax collection and justice functions among others. Although its educational provision has been characterized as weak, the municipality does provide basic and higher education, such as university programmes, to the region [24]. Health service infrastructure is also an important characteristic of Girardot’s regional primacy, including a high-complexity (3rd level) hospital that serves as a departmental referral centre [25].

According to official records, approximately 23% of reported dengue cases are residents from other municipalities, including the non-dengue-endemic settlements of the Andean region that are located at higher than 1800 MASL, where the dengue vector is absent (e.g., Bogotá). These settlements provide a constant flux of people who are susceptible to the disease. Although a precise estimate does not exist, Girardot and its neighbouring touristic municipalities receive approximately 65,000 visitors in a regular weekend, approximately 140,000 over long weekends and up to 300,000 tourists during high season holidays (June–July and December–January) [24].

As stated before, Girardot is in the central region of the country where important movements of people generally occur. This central region is characterized as having both hyper-endemic transmission (i.e., the persistent transmission of dengue, with the simultaneous circulation of the 4 dengue serotypes) and endemic-epidemic transmission (i.e., sustained dengue transmission with punctual epidemic outbreaks). Girardot is responsible for 30.9% of the reported cases in the Department of Cundinamarca, and it is one of the 18 municipalities (*n* = 1112) that accumulated 50% of dengue cases in Colombia (1999–2010) [8].

Girardot is a growing tourism-oriented city with an influence over surrounding municipalities including non-endemic cities such as Bogotá. Additionally, Girardot is situated in a hyper-endemic region for dengue transmission. Although urban growth and tourism have been considered as important factors of dengue transmission elsewhere [4, 19, 26, 27], insufficient evidence is available about these factors for Colombian cities.

## Methods

### Sources and treatment of information

Information sources and the processes applied to perform a spatial analysis are described below. The obligatory case report database from 2012 to 2015 (SIVIGILA), block population information (Census 2005) and meteorological information (temperature and precipitation, 2012–2015) were used.

#### SIVIGILA

In Colombia, dengue cases are reported through the national public health surveillance system (SIVIGILA). Cases are identified and reported by the healthcare system, classifying the event as either probable dengue or probable severe dengue (depending on clinical observation) [2, 28].

The challenges of using this type of information have been discussed in Colombia [29] and in other countries [15, 30, 31]. Reported cases correspond only to symptomatic patients who present at healthcare centres, and they are registered with their residence addresses that are not necessarily the places of transmission. In addition, the SIVIGILA database does not contain specific serotype infection information.

**Fig. 1:**
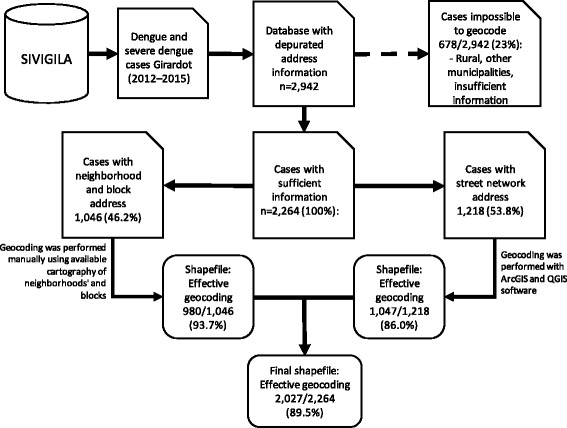
Flowchart of the geocoding process

The geocoding of dengue cases in Girardot is challenging because the address information in the database is not standardized, and two different systems of urban addresses coexist in the city. This issue was resolved via the intensive depuration of the database and combining different automatic (ArcGIS Desktop: Release 10. Redlands, CA: Environmental Systems Research Institute, and Quantum GIS: Open Source Geospatial Foundation Project) and manual geocoding strategies, assuring geocoding precision at the block level (Fig. 1). The results of the geocoding process are not usually shown in detail; nevertheless, they are crucial to evaluate for possible selection bias. Figure 1 shows the results of the geocoding process. Of the 2942 cases in total, 23% were classified as impossible to geocode. The majority of these cases corresponded to dengue cases with a residence address from another municipality (406). Others had insufficient information for geocoding (228). Although dengue is described as a primarily urban disease [32], 44 cases were associated with rural addresses that were not possible to geocode. Of the 2264 cases with sufficient information, 89.5% (2027) were successfully geocoded for spatial analysis. A visual inspection of the geocoded cases using satellite images from Google Earth revealed an even distribution of cases throughout the residential areas of the city.

### Intra-urban census data

An important issue to consider when performing these types of spatial analyses is the distribution of the population, which might act as a confounding factor when assessing the distribution of dengue cases.

Total population per block data were available from Colombia’s last population census (2005), with a total population of 87,626 across 1197 polygons that cover the urban area. Although the available spatial information is not recent, it remains an important dataset that provides a relative measurement of the population distribution to assess the distribution of dengue cases. Acknowledging that the spatial population information is from 2005, and the dengue cases assessed are from a later period (2012–2015), a ratio of dengue cases per inhabitants at the block scale was used, understanding that it provides a relational view that enables the development of a spatial analysis more than an accurate measurement. Another study undertook a similar relational approach [7].

Using Google Earth’s satellite images from multiple years, spatial information (i.e., blocks) was adjusted for non-residential areas (parks, undeveloped areas, and other land uses), excluding polygons without population information (225) and those without residential areas (7). The final result was a shapefile with 965 polygons for a total population of 86,460 inhabitants.

The block polygons have a mean area of 8185 m^2^, although with a wide range (222 to 274,296 m^2^ polygons). Given the difference in the size of the polygons that contain population data, a second approach was developed to enhance the spatial analysis possibilities. Local cluster analyses based on centroids of polygon information are sensitive to the number and distances between neighbouring data points. To resolve this situation, Telle [15] used a grid with squares of 250 m covering the study area (Delhi, India) to reduce the effect of irregular spatial units (neighbourhoods). In this example, satellite images were used to estimate the population of each square. Using a similar approach, a 20 × 20-m grid was created to represent population information, dividing the block’s population by the number of squares contained in each block. Importantly, this procedure assumed that the population was evenly distributed inside each block polygon.

This procedure is also appropriate for Girardot because the population density within blocks is similar, and houses are the predominant type of lodgement in residential areas. In the central region of the city where small buildings (with a higher population density) are present, blocks tend to be smaller; thus, the applied procedure does not have an important effect. For both approaches, 1994 of the 2027 geocoded cases were within the block polygons and grid squares that contained population information.

### Meteorological data from field stations (2012–2015)

Finally, precipitation information was obtained from the Hydrology, Meteorology and Environmental Studies Institute of Colombia (IDEAM). Two datasets were used for this study. The first included the monthly mean precipitation for a long time period (1981–2010) from 5 meteorological stations located near Girardot’s urban area. This information provided the general behaviour of the precipitation in the region. A more specific dataset was obtained from a nearby station (located in the neighbouring municipality of Flandes) of the monthly total precipitation during the 2012–2015 period. This information enabled an approximate assessment of the precipitation behaviour during the same study period in which the dengue cases were evaluated.

### Spatial analysis

Spatial autocorrelation enables the assessment of whether the distribution of a given phenomenon corresponds to a specific spatial pattern (e.g., aggregation or dispersion) or whether it resembles a random distribution. These methods generally provide a global measure used to assess the aggregation of data within a dataset and a local measure to evaluate the location of the clustered data. For this study, Getis-Ord indices (both global and local) were used [33].

The dengue cases were divided into six different datasets (Table 1) for the spatial autocorrelation analysis. The first 4 datasets corresponded to individual year datasets (2012, 2013, 2014 and 2015), whereas the other 2 datasets were generated based on the rainy and dry seasons for the entire study period (2012–2015; rainy seasons, March–May and October–November). For each dataset, cases per inhabitant were calculated following the two approaches described above (block polygons and grid). Finally, evaluating the urban morphology of Girardot, non-residential use areas fragmented residential areas, and isolated constructed areas were identified west of the municipality. For this reason and after conducting different tests, it was decided that a fixed band of 300 m would be used for a spatial correlation analysis at the block scale, and a fixed band of 100 m would be used at the grid scale. These distances ensured that all of the data would have at least one neighbour (even between fragmented residential areas); furthermore, it corresponded to the mobility range of mosquitoes, which is important for the diffusion process of the disease (human-vector-human). Another study considered similar distance ranges as adequate for this type of analysis [15].

**Table 1:** Results of global indices using block centroids and a 20-m grid

Dataset	Number of geocoded dengue cases (*n* = 1994)	General Getis-Ord 20-m grid (Confidence level)	
		Fixed band 300 m	Fixed band 100 m
2012 dataset	380	99%	99%
2013 dataset	723	99%	99%
2014 dataset	533	99%	99%
2015 dataset	358	Not significant	Not significant
Rainy season (2012–2015)	732	99%	99%
Dry season (2012–2015)	1262	99%	99%

### Kulldorff’s scan statistics

Kulldorff’s space-time scan statistics were used to assess the concentration of dengue cases [34]. Performing a gradual scan with variable-sized windows over a determined space-time, cases inside each possible window were noted and compared with the expected cases for the same window. To accomplish this goal, information regarding the population at risk in each location was required. After detecting the most likely space-time clusters (primary and secondary), these clusters were tested via Monte Carlo simulations: If the points conforming to the evaluated cluster maintained their aggregated pattern when compared with 999 randomized simulations of the entire dataset, then it was considered significant. Unlike spatial autocorrelation methodologies (e.g., Moran’s or the Getis-Ord index), scan statistics enable the assessment of both the aggregation of observations and the location of the aggregated observations that reject the null hypothesis via a Poisson model: Expected cases are proportional to the population size [34, 35].

In vector-borne diseases (and specifically in dengue research), this type of analysis enables a better understanding of the disease’s spatial and temporal dynamics, correlating cases not only with regard to proximity but also within a plausible period during which human-mosquito-human transmission might occur. Other studies have used this analysis to assess the configuration of outbreaks, identify concentrations of cases in vulnerable populations and improve decision-making processes [36–39].

### Information and parameters used for the space-time analysis

The same information used for the autocorrelation analysis was used for this approach to analyse individual years (2012–2015). Different dates for each dengue case were registered in SIVIGILA’s database (notification, onset of symptoms, hospitalization, and so on). Because of the spatial and temporal nature of the analysis, the symptom onset date was selected because it is a better proxy for the actual infection date than the case notification date. Dengue cases and population information at the block level were used for this analysis (965 spatial units). A finer scale analysis, such as the grid used for autocorrelation processing, was not possible because of the calculation time required to scan the more than 17,000 spatial units that conformed to the grid.

An advantage of the software selected to develop the analysis, was its recognized ability to customize model parameters [40]. In this case, a retrospective space-time design was used based on a discrete Poisson probability model. A time-segregation period of 20 days was selected, and 50% of the at-risk population was set as the maximum spatial cluster size. Nevertheless, only clusters with radii smaller than 300 m were reported. A supplemental file contains details of the used parameters (Additional file 1).

## Results

**Fig. 2:**
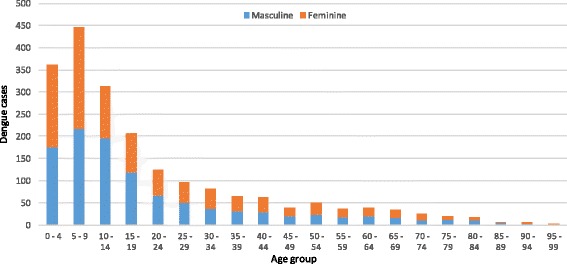
Dengue cases by age and sex, Girardot 2012–2015 (*n* = 2027). Source: SIVIGILA records (adjusted for only local residents)

Geocoded dengue cases (*n* = 2027) were slightly overrepresented by men (52.1%), although important differences were registered for the 10- to 14-year-old and 75- to 89-year-old age groups, in which men showed a higher degree of participation (greater than 60%). By contrast, the 70- to 74-year-old age group reported 60% of cases in women (Fig. 2). Dengue cases in Girardot predominated in the first 15 years of life, as expected according to known trends for Colombia [41].

**Fig. 3:**
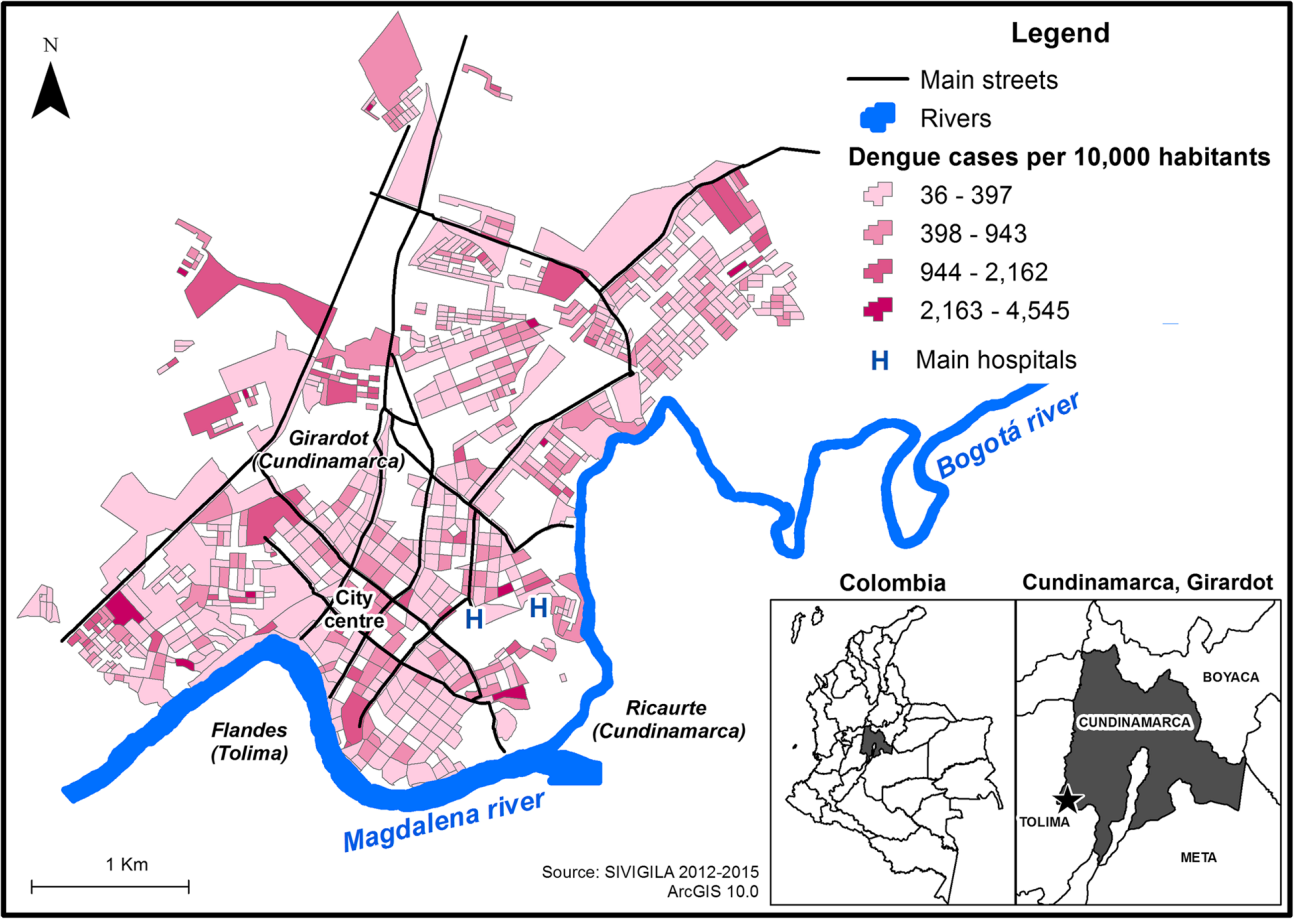
Incidence of dengue in the context of residential urban morphology, Girardot 2012–2015

The incidence of dengue is represented on Fig. 3. Near the bank of the Magdalena River, two concentrations of cases were observed (East and West), as in the northeast and northwest limits of the urban area. On the map, the urban morphology of residential areas is appraisable, showing a north/south division of non-residential land use (university campuses, undeveloped areas, sport complexes) connected by main streets at three points. Block shape, extension and orientation differed and were fragmented by open areas (undeveloped terrain and parks), although they were well connected by main streets.

The conditions of the residential areas were also heterogeneous and unevenly distributed throughout the urban area, displaying a slight pattern of new construction (corresponding to different socioeconomic strata) along the western corridor, whereas low socioeconomic strata residences tended to concentrate along the banks of the Magdalena and Bogotá Rivers, and more formal habitation was located towards the city centre. Nevertheless, no defined or general residential pattern was observed based on socioeconomic conditions, with a mixture of closed residential developments (second residential housing) and formal and informal residence areas.

### Persistence of dengue cases in Girardot (Getis-Ord)

Using the population grid approach, the global Getis-Ord index was significant for the rainy and dry seasons as well as for 2012, 2013 and 2014. Only the 2015 dataset was not eligible for further local exploration because it was not significant for the global cluster analysis (Table 1). A local Getis-Ord was applied for the incidence of dengue using a 20-m grid with fixed bands of 300 and 100 m. Both results were consistent, showing a similar distribution of clustered dengue cases; however, the result with a 100-m fixed band allowed a more precise representation of clusters in the context of Girardot’s fragmented residential areas.

**Fig. 4:**
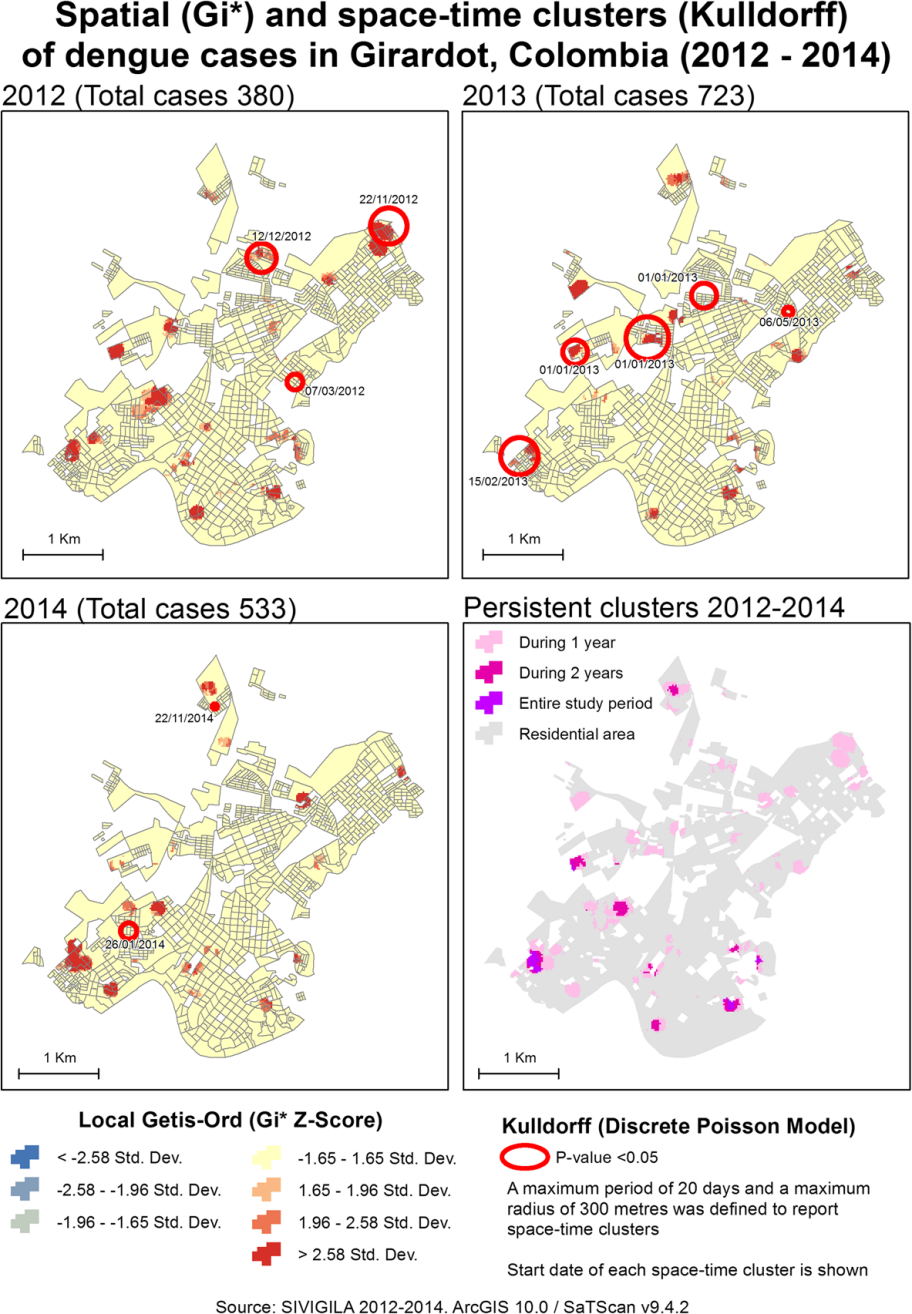
Spatial distribution of the incidence of dengue in Girardot

Figure 4 shows the local distribution of the incidence of dengue clusters for the 3 years that were significant for global tests: 2012, 2013 and 2014. Interestingly, the concentration of dengue cases differed by year and was not always related to the number of cases. In 2012, 380 cases were reported; however, from a spatial perspective, these cases configured more clusters than the subsequent years, although they reported more cases (723 in 2013 and 533 in 2014).

In terms of geographical extension, 2 clusters were notable in 2012, corresponding to different neighbourhoods. At the northeast limit of the urban area, an extent cluster covered part of Portachuelo, a peripheral neighbourhood with formal and informal housing. A second large cluster was located in the central-west region of Girardot, where more heterogenic urban conditions are present: Quinta Saavedra has formal but diverse residential areas (middle-low socioeconomic conditions and second residence complexes); El Portal de la Hacienda is a closed secondary residential area; and part of Joge Eliecer Gaitan, a neighbourhood also characterised by middle-low socioeconomic conditions.

Regarding 2013, an important cluster is visible in the southwest region of the city, primarily involving La Esperanza, Hacienda Girardot and Quinto Patio, which share similar middle socioeconomic conditions, and specific areas with more vulnerable conditions. This cluster also expands to the other side of the main corridor that connects the north and south of the city, where Condomino Los Angeles is located, an area with affluent secondary residence complexes.

The persistence of clusters throughout the study period is represented in the bottom right-hand corner of Fig. 4. A general pattern of more persistent clusters (present in 2 of the 3 years of the study period) is notable in the south of the city, which contrasts with the north, where clusters were apparent only for 1 year. This northern area also has a more fragmented residential morphology. Nevertheless, two persistent clusters were identified at the southwest and southeast limits of the urban area. The first affects La Esperanza, Hacienda Girardot and Quito Patio as described above, whereas the second affects Las Rosas and part of Las Bocas del Bogotá. In general, these latter neighbourhoods have lower socioeconomic conditions, including isolated informal residences.

**Fig. 5:**
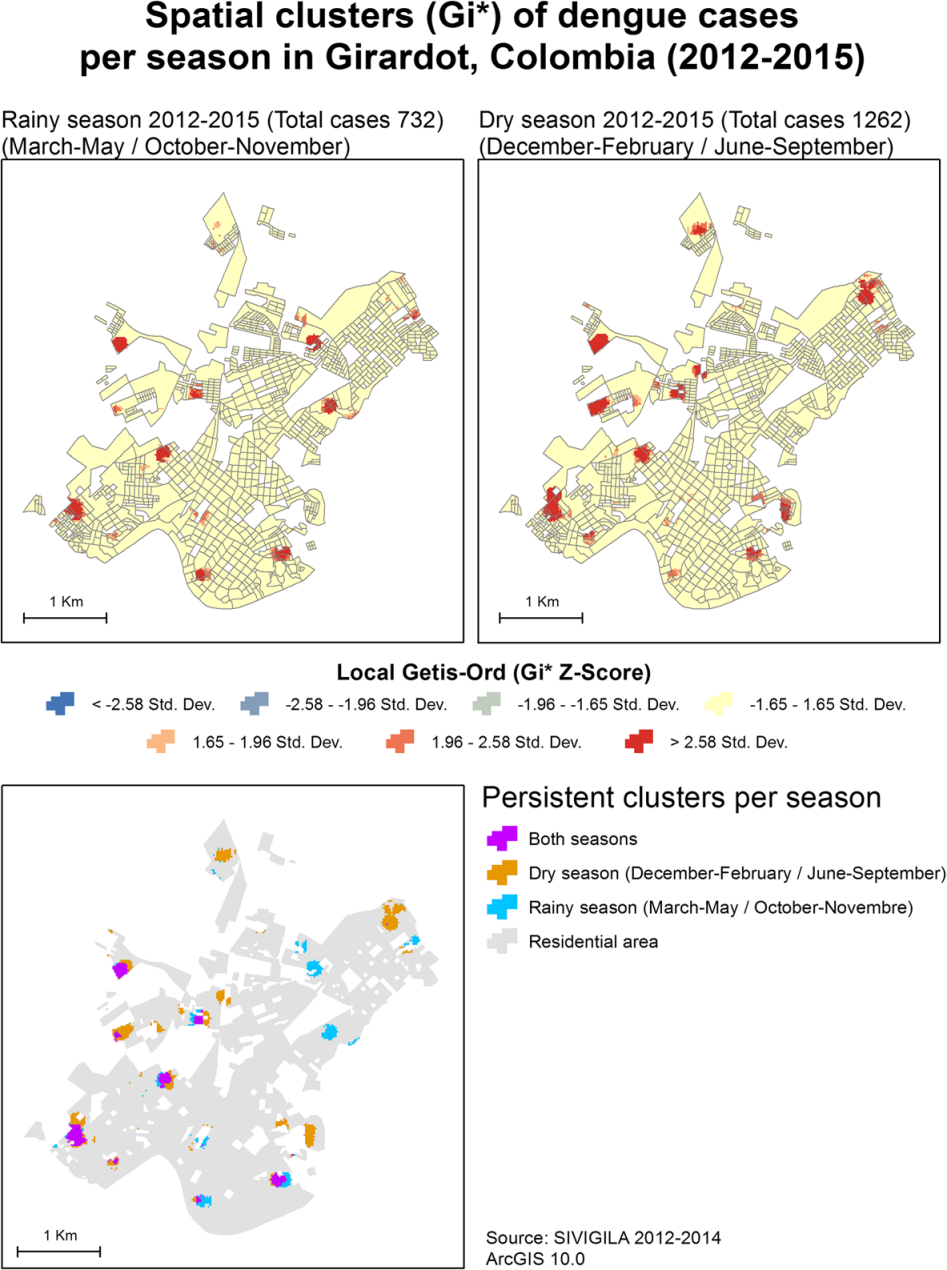
Seasonal distribution of the incidence of dengue in Girardot

Figure 5 shows a general pattern describing a diagonal, where clusters with persistent presence in the dry and rainy seasons are located in the south and west regions of the urban area. Included in this group are both of the persistent clusters identified earlier for the 3 years studied (2012–2014). At the other side of this diagonal, clusters with a presence in only one of the seasons during the evaluated period (2012–2015) were located in the north and east regions of the city. Neighbourhoods where significant clusters were present for the rainy or dry season differed with regard to socioeconomic conditions, ranging from informal developing areas to formal and closed secondary residential areas with high socioeconomic conditions.

Importantly, the number of cases per season differed: 1262 in the dry seasons and 732 in the rainy seasons. Although the dry season includes 7 months (December–February and June–September), and the rainy season includes only 5 (March–May and October–November), the former still had a higher mean number of cases per month (180.3) than the latter (146.4). It is unlikely that this difference is due to under-report of cases in different periods. As stated earlier, there is an increase of tourism (grater presence of susceptible populations, higher population density) during the dry seasons that could have an effect on the increase of dengue cases.

Table 2 shows the percentages that the grouped cases in significant clusters represent. Although the percentages were generally low, the spatial distribution of these clusters corresponded to different neighbourhoods as discussed above. Regarding patient age, Table 3 shows that all of the clustered cases were associated with lower mean and median ages than non-clustered cases, although no significant differences were found using a nonparametric analysis (K-sample test on the equality of medians, StataCorp. 2013. Stata Statistical Software: Release 13. College Station, TX: StataCorp LP).

**Table 2:** Cases grouped in significant clusters

Year/season	Total cases	Cases grouped in significant clusters(*p* < 0.05)	%
2012	380	66	17.4
2013	723	101	14.0
2014	533	50	9.4
Any of the 3 years	1636	325	19.9
Persistence in at least 2 years	1636	91	5.6
Rainy season	732	63	8.6
Dry season	1262	141	11.2

**Table 3:** Age of the patients in the identified clusters

	% of cases younger than 15 years old	Mean age (years)	Median age (years)	n	Pearson’s chi^2^/*P*-value (K-sample test on the equality of medians)
Cases in persistent zones (clusters in at least 2 years)	70.3	16.6	11	91	3.2333/0.072
Cases in non-persistent zones (clusters in at least 2 years)	60.4	19.4	12	1545	
Cases clustered in the dry season	60.3	17.1	12	141	0.8484/0.357
Cases not clustered in the dry season	58.0	20.1	13	1121	
Cases clustered in the rainy season	68.3	18.1	10	63	1.4416/0.230
Cases not clustered in the rainy season	57.2	21.2	13	669	
Cases clustered in any of the three years	64.3	17.4	11	325	3.6110/0.057
Cases not clustered in any of the three years	60.1	19.8	12	1311	

### Concentration of cases in time and space (Kulldorff’s scan statistics)

Figure 4 represents the 10 spatio-temporal clusters identified from 2012 to 2014. Examining the start dates of the clusters, they shifted starting on 22 November 2012 in the northeast (Portachuelo) towards the southwest (La Esperanza). The established 20-day window of the first cluster ended on 11 December, and three new clusters started on the next day (12 December) with the separation of 1230, 180 and 457 m among them, ending on 25 January 2013. A fifth cluster started 20 days later (15 February) in the southeast region of Girardot.

The identified clusters also described the 2013 outbreak, with three initial clusters during the first year (2012), increasing to 5 in 2013 and then decreasing to two clusters in 2014. As shown in Additional file 2 (spatio-temporal cluster detail), 5 of the 10 spatio-temporal clusters overlapped with spatial clusters (Getis-Ord) that represented a concentration of cases during each year. The spatio-temporal clusters also varied in size, reporting radii from 33.5 to 266.9 m. Different types of residential areas and urban conditions were reported, including opposite conditions such as in the Agua Blanca and Brisas de Guadalquivir high-stratum residence complexes, which were different to the vulnerable conditions in Portachuelo. Within the presence of these two extremes, average conditions were constantly reported.

During the 3-year period, spatio-temporal clusters included 69 observed cases, corresponding to only 4.25% of the total cases reported over the same period (1623 cases). These cases were primarily patients younger than 15 years old, with an even distribution between males and females (Additional file 2).

**Fig. 6:**
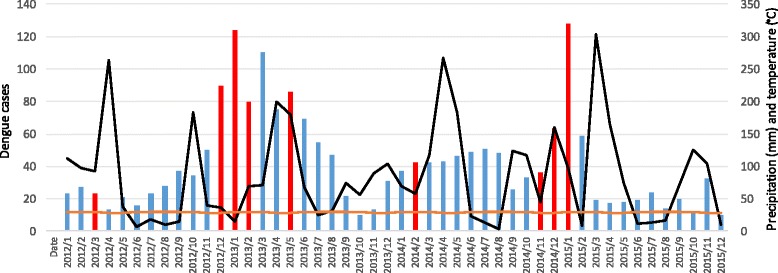
Dengue cases, temperature and precipitation, Girardot 2012–2015. Legend. Black and orange lines along the right axis represent the total precipitation (mm) per month and temperature (°C), respectively. The bars represent dengue cases per month. The highlighted bars in red indicate the presence of spatio-temporal clusters. Sources: SIVIGILA and IDEAM

Regarding dengue cases on a broader temporal scale, an increased number of cases was noted at the end and beginning of each year, especially for 2012–2013 and 2013–2014 (Fig. 6). For both epidemic periods, spatio-temporal clusters were identified (red bars in Fig. 6) during the first months when the increase of cases occurred. As mentioned earlier, December–February and June–September are the dry seasons. However, to assess specific interactions of precipitation with dengue cases, the black line in the same figure represents the total monthly precipitation in mm, showing that dengue cases tend to increase during dry seasons.

**Fig. 7:**
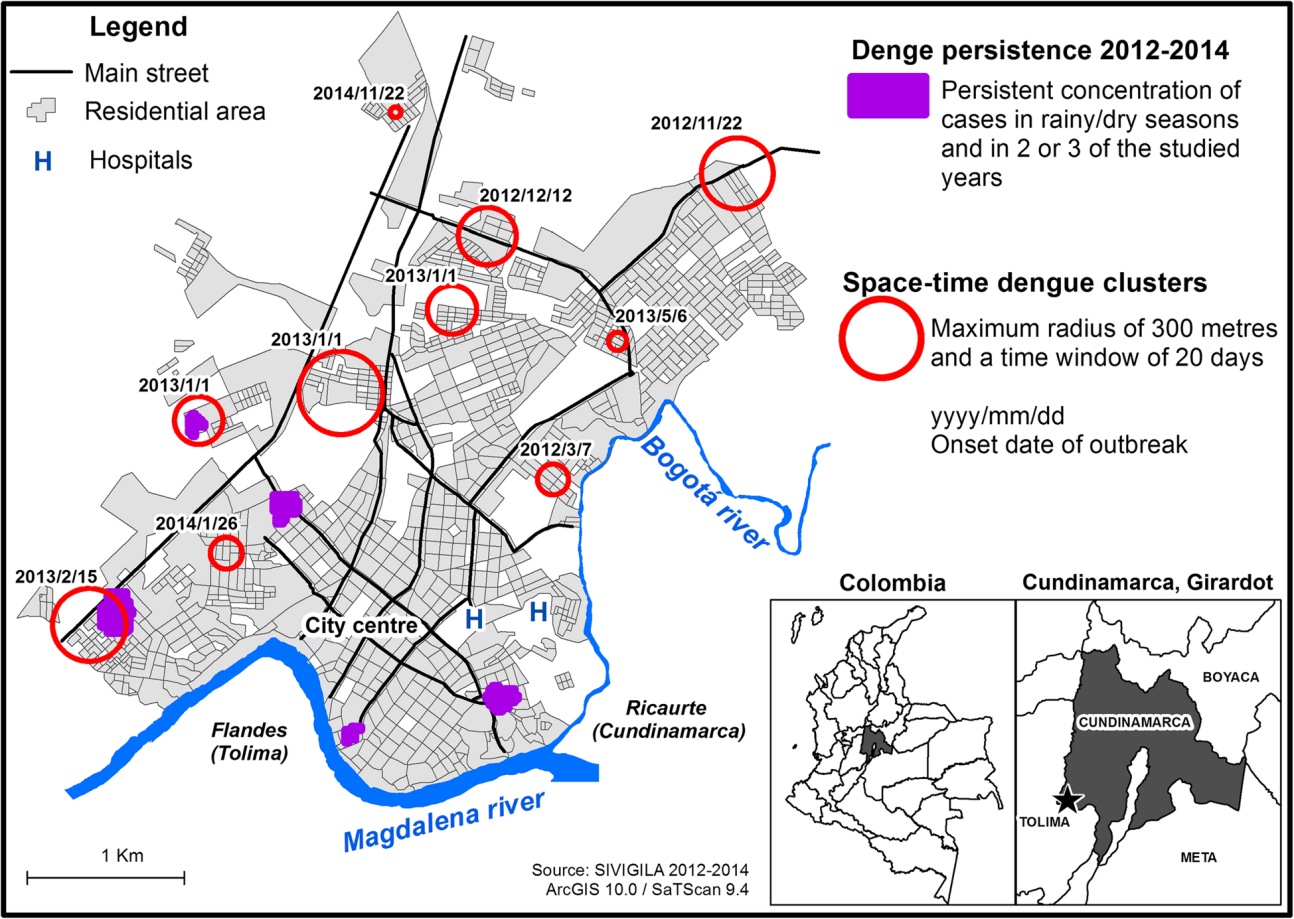
Synthesis of the incidence of dengue in Girardot

According to the synthesis (Fig. 7), dengue cases clustered in space and space-time described before showed different distributions. A persistent incidence of dengue (local Getis-Ord indices) was found in more consolidated urban areas, whereas the space-time clusters (Kulldorff’s scan statistics) emerged towards the north and east of the city where urban expansion and a more dynamic urban space are present. Nevertheless, dengue clusters assessed with both methods were located outside of the central region of the city towards the periphery, corresponding to the neighbourhoods and residential areas with different characteristics and socioeconomic conditions. The different distributions of space and space-time clusters could be related to different territorial dynamics as discussed in the next section.

## Discussion

This study combined spatial and spatio-temporal analyses, which have been proven useful [42] for assessing the distribution of the incidence of dengue in intra-urban settings. Although the observed clusters in Girardot shifted throughout time and space (Fig. 7), these patterns might be the result of different territorial dynamics related to urban functions, urban changes (i.e., urban expansion), demographic composition, or touristic activities. Further understanding of dengue persistence (space clusters) and outbreaks (space time clusters) in the context of hyper-endemic urban spaces could eventually help to guide prevention measures from a territorial perspective (i.e., where, when and how to intervene).

### Space clustering of the incidence of dengue

The geography of dengue in Girardot is characterized by annual concentrations of cases in clusters of different sizes across disparate places throughout the city. Similar results were obtained in a study in Delhi [15], where a similar number of cases were assessed over 2 years. The results showed concentrations of cases in different locations. The author emphasized that a system in apparent equilibrium (i.e., the same number of cases in two consecutive years) might hide an unstable configuration of the incidence of dengue on a finer scale. Although the number of cases in Girardot changed over years, similar numbers were reported in 2012 and 2015 (380 and 358, respectively). Nevertheless, the results for the general Getis-Ord index showed that the cases in 2015, unlike 2012, did not correspond to an agglomerated pattern; thus, assessments of local clustering for this particular year are not recommended. Even more drastic that the results from Delhi, the similar count of dengue cases in Girardot might not be significantly concentrated.

Nevertheless, this study described the persistence of significant concentrations of dengue cases, defined as the overlapping of clusters for 2 or 3 years of the study period as well as in both seasons (Figs. 4 and 5). A 3-year study in another hyper-endemic urban context [16] found a low percentage of the spatial units registered dengue cases in 2 or 3 of the study years. Therefore, an absence of permanent clusters was described in the context of a highly populated city (Delhi) undergoing the reintroduction of the DENV-1 serotype 5 years after its last circulation. The overlap of significant clusters described for Girardot accounted for a small percentage of the total cases for each year or season (Table 2); nevertheless, this persistence was observed despite the local effect of monotypic herd immunity, which can weaken the persistence of the incidence as described by Telle et al. [16]. Moreover, these clusters showed a distribution that was coherent with the local epidemiology of dengue and residential structures.

Non-significant median age differences were found among patients in clustered and non-clustered patterns in Girardot (Table 3). However, the higher percentage of cases in patients younger than 15 years old and the lower mean age of clustered cases supports the hypothesis that the presence of vulnerable populations shapes the distribution of the identified clusters, given that the most affected age group in Colombia shifted from 15 to 44 years old towards 0 to 15 years old [41]. This result is a response to the actual hyper-endemic transmission in cities such as Girardot and characterized by the simultaneous circulation of all four dengue virus serotypes observed since 2004. A multi-country study in Asia and Latin America (5 countries in each region) assessed symptomatic dengue and seropositivity in children [43]; Colombia contributed the largest subcohort of the region (*n* = 3245) distributed among 9 municipalities, including Girardot. Although non-specific data were available for Girardot, Colombia reported the highest seropositivity at baseline (92.3%) in Latin America, and it was the only country of the region to report the circulation of all 4 dengue serotypes. Additional studies are needed to assess the impact of demographic composition at a fine scale on the incidence of dengue in the context of hyper-endemic transmission.

Regarding the distribution in the residential structures of the described persistent clusters, three of the five areas that showed this persistent behaviour corresponded to neighbourhoods of local residents (non-tourists) with low-to-medium socioeconomic conditions; one corresponded also to neighbourhoods of local residents with heterogenic conditions but with a predominance of medium socioeconomic conditions; and finally, one was related to a high stratum of secondary residence complexes (Brisas de Guadalquivir). Although the available information for this study did not enable an assessment of the specific socioeconomic conditions of each of the five mentioned areas, a relationship between the structural socioeconomic conditions and the persistence of dengue is likely. A previous study of Girardot [44] that evaluated dengue cases (1998–2002) from an ecological approach obtained results consistent with those discussed here. Using a standardized incidence ratio (i.e., the incidence of dengue for each neighbourhood divided by the incidence of dengue for the whole city), a substantial number of neighbourhoods were identified at high risk [44], including the same specific areas of persistent clusters discussed earlier (excluding the high-class complex Brisas de Guadalquivir, which did not exist at the time). Furthermore, a general relationship was found between high-risk neighbourhoods with low socioeconomic status and the presence of the vector in households. However, these general findings were not suitable for assessing specific intra-urban dynamics.

A systematic review argued that there is no consistent evidence to affirm that poverty is a predictor of dengue [13]. This lack of evidence might also be related to the scale of analysis usually adopted to assess this type of relationship. Considering the persistence of dengue at an intra-urban scale might help define a more appropriate scale to measure the relationship between dengue and socioeconomic conditions, which might also help explore how other systems interact to explain why not all deprived areas have concentrated cases or why dengue cases are also concentrated in high-stratum areas such as Brisas de Guadalquivir in Girardot. Nevertheless, we agree with other authors [7] that this type of finding should strengthen rather than discourage public policy to attain sustainable urban development, including dengue prevention and control programmes, especially for those with greater need.

Olivier Telle [15] found that intra-urban deprived and densely populated spaces in Delhi were associated with dengue concentration and the permanent presence of the dengue vector throughout the year. This relationship might play an important role in maintaining viral circulation during inter-epidemic periods. The degree of vulnerability of deprived urban areas and their relationships with vector presence have yet to be adequately measured in Girardot. Nevertheless, the persistence of the incidence of dengue was found near the Bogotá and Magdalena Rivers and in the oldest, more consolidated and less fragmented regions of Girardot. This finding contrasted with the northern part of the city, which presented with a more fragmented residential morphology, an important commercial and tourism-related infrastructure (e.g., shopping malls), and the two urban expansion areas of the city (i.e., the northern and western fronts).

Related to the urban morphology of the city, dengue clusters identified during the rainy season (Fig. 5) were located near small natural drainages (caños) or open, undeveloped terrain. Although the vector productivity in Girardot has been primarily related to indoor recipients [45], this result suggests that specific places exist where the rainy season increases the productivity of breeding sites in public spaces and therefore augments the risk of dengue and the number of cases. This suggestion is a plausible hypothesis, given that undeveloped terrains and “caños” are usually unattended and filled with rubbish, creating a potential accumulation of rainwater and consequent breeding sites for *Aedes aegypti*. Major breeding sites in public spaces (specifically sewers), have been reported in other Colombian cities such as Cali [45].

One overall point about the persistent behaviour of dengue concentrations is that it seems to be related to low and medium socioeconomic conditions. In addition, this persistence was correlated with more consolidated and stable urban systems, unlike the north and west expansion areas of Girardot.

### Space-time clustering of the incidence of dengue

Regarding the results of the spatio-temporal analysis, the general spatial distribution differed from the pattern discussed regarding persistent agglomerations of the incidence of dengue. Outbreaks have been described along a diagonal from the northeast towards the southwest of the city, following the axis of urban expansion described earlier, whereas a persistence of cases was present in the southern part of the city. However, common places remained (5 of the 10 spatio-temporal reported clusters) where these two distributions overlapped, specifically throughout the onset and initial evolution of the 2013 epidemic (November 2012–February 2013).

However, the observed cases within the space-time clusters represent a small percentage of the total reports (4.25%). This result diverges from that obtained by a similar analysis that assessed a specific outbreak with the same measurement, finding 65.3% of total dengue cases [46]. Another study (with the same model parameters: 300 m and 20-day windows) observed 67% and 62% of the total cases in spatio-temporal clusters over two consecutive years with similar numbers of cases [15]. In the context of hyper-endemic dengue transmission in Girardot, cases were distributed throughout the city year-round. Therefore, space-time clusters might represent particular transmission dynamics more than focalized starting points for generalized epidemics. Nevertheless, 68% of the cases grouped in space-time clusters were located in areas with tourist infrastructures and secondary residences; furthermore, they occurred during the dry season, which is the longest and most tourism-related period of the year in Girardot (December and January).

This temporal distribution contrasts with the described relationship during the rainy season and the increase in dengue cases in Colombia [4, 41]. In Girardot, a clear pattern of dengue cases is notable during the dry season. The results of an entomologic study support this finding, stating that Girardot reported (in 20 randomly selected clusters composed of approximately 100 households each) more recipients with water, a slight increase in low tanks (albercas) with high water levels and an overall increased productivity of *Aedes aegypti* pupae during the dry season [47]. Importantly, 19.6% of 3228 reported cases in the initial processing of the SIVIGILA database (2012–2015) corresponded to rural residents and residents from other municipalities. This finding led to the perception of important city migration fluxes due to tourism, low-wage construction workers and population from surrounding municipalities in search of services. These migration dynamics are commonly reported for Girardot but have not been well documented or measured.

The spatio-temporal clusters also describe the displacement from the northeast (November 2012) to the southwest (February 2013). In another study, the possible effect of wind was discussed, which was associated with the dispersal of dengue epidemics [46]. In this case, however, it seems that social structures and the migration of susceptible populations remain as possible explanations. The onset date (1 January) of three simultaneous space-time clusters might be correlated with the long tourism season of the city because two clusters overlap with high-stratum secondary residence complexes. One possible hypothesis is that the arrival of susceptible populations to secondary residence complexes during a season when the vector densities are higher [47] results in conditions conducive to the spread of the virus, thereby generating symptomatic reported cases as well as asymptomatic infections that generally affect locals and foreigners. Although the influence of tourism has been explored in other studies [4, 19, 27], more evidence is needed for Colombian cities such as Girardot. Findings concerning touristic economies and risk of outbreaks are sensible and have an important effect in local economies, as reported in the Indian Ocean during the chikungunya outbreak (2004–2006) [48]. In the case of Girardot, recent outbreaks of chikungunya and Zika have concerned local authorities regarding health and economic impacts (mainly in tourism).

To attain a better understanding of the possible transmission dynamics relative to the interactions between ecological and social systems, much remains to be learned about the intra-urban circulation of dengue serotypes and their relationships with the movement of people (at different scales), especially with regard to Girardot and Colombia. Much has been said about the effects of daily movements, including the methodological challenges regarding accurate measurements and the estimated effect of daily movement on dengue transmission [7, 30, 31, 49–51]. Intra-urban daily movements have been suspected in hyper-endemic cities such as Girardot, given the low percentage of cases represented in spatio-temporal clusters. Assessing cases by their residential address can affect a spatio-temporal analysis, given that people might be infected away from home. Additional evidence is needed to understand how daily movements shape dengue transmission in Girardot.

### Dengue in Girardot: Among places of persistence and places of outbreaks

The incidence of dengue in Girardot was characterized with space and space-time manifestations throughout an urban space. Each cluster had different spatial patterns; however, a general pattern was evident, in which persistent clusters (local Getis-Ord indices) and space-time concentrations (Kulldorff’s scan statistics) occurred along the periphery of the city, identifying a weak presence of the disease in the historical and geographical centre of Girardot. Although the incidence analysis controlled for the effect of population density, spatial units with low population denominators (which are usually present in city centres) can overestimate the incidence, even when they present a reduced number of cases [15]. This finding was not the case for Girardot, even that population density is lower in the city centre because of other non-residential land uses; nevertheless, no apparent dengue concentrations were detected.

In other contexts, the city centre has been described as having a high incidence of dengue [7, 52]. However, peripheral vulnerable areas have also been emphasized to play a role in understanding dengue transmission, and interventions are recommended in these places even if incidence of dengue is low [7, 15]. The periphery of Girardot was not specifically correlated with vulnerable conditions because of the mixed residential pattern already described. Nevertheless, the peripheral spaces in Girardot are dynamic, and they demonstrate the recent urban changes that the city has undergone in terms of housing construction and commercial/tourism activity emplacement. In contrast to dengue persistence, spatio-temporal clustering seems to be related to areas where seasonal fluxes are present, specifically with regard to susceptible populations. In the same sense, dynamic and changing urban spaces (expansion and changes in urban functions) were related to the spatio-temporal concentrations of the incidence of dengue.

Complementary use of space and space-time analysis can reveal different underlying territorial dynamics. Structural or long-term urban conditions (e.g., deprived areas) might be related to persistent concentrations of dengue to a certain extent. By contrast, more unstable and temporal urban dynamics (urban expansion and the movement of people on different scales) might enhance spatio-temporal concentrations. This possible pattern would certainly need to be evaluated in the context of and particularities of specific urban spaces before arriving at generalized conclusions.

### Study limitations

Working with notified dengue cases (SIVIGILA) posed a series of challenges mentioned throughout this paper. The absence of serotype information related to reported dengue cases limits the interpretation of the space and space-time clusters described in this study. One of the most important limitations concerning this type of study is the accuracy of address information in Colombia, which required an important and comprehensive process of database editing. In addition, differential access to the healthcare system (in which cases are officially reported) might have underestimated the incidence of dengue among specific social groups (e.g., inhabitants from more deprived areas). Finally, no evidence exists as to whether the distribution of reported symptomatic cases is comparable to the asymptomatic non-registered cases; if important differences exist, then the results might be biased.

Context-related limitations were also present, such as the availability of updated populations and socioeconomic information, particularly with regard to the detailed spatial units that enabled additional exploration of the distribution of cases in urban areas. Population denominators are crucial for this type of analysis, and although approximate results can be achieved using the available information, having more accurate data that can lead to better results and more useful recommendations is always desirable.

## Conclusions

The literature on dengue is full of inconsistent results that mirror the inherent complexity of its transmission dynamics. The relationships between socioeconomic conditions and the incidence of dengue have not been completely unveiled. In addition, the movement of people and the specific circulation of serotypes on fine scales are being explored as important components of dengue transmission. Finally, although the introduction of dengue vaccines is on the horizon [53, 54] and much knowledge has been gained regarding this disease, many questions remain to be addressed to advance effective prevention initiatives to affect its burden. From epidemiological, public health and social sciences viewpoints, the need for coordinated collaboration that leads to assessments of the effect of “place” from a relational perspective is gaining importance [55], and geography has an important role to play.

A general peripheral pattern was described in Girardot; however, by no means is exclusively correlated with vulnerable spaces. Rather, an understanding exists that the periphery of the city is marked by the presence of different types of residential areas that correspond to a wide range of socioeconomic and cultural conditions. From a geography of health perspective, it is important to acknowledge that the linear and general associations between dengue and poverty or peripheral spaces no longer apply specifically to intra-urban hyper-endemic settings. The pathogenic complex of dengue [18] is dynamic, and even if the same main components are always present (virus, vector and susceptible population), the configuration of these components with regard to complex urban territorial structures would result in different processes and spatial manifestations of the disease.

## Additional files

Additional file 1: Parameters for space-time cluster analysis (SaTScan V9.4.2). Description of data: Table containing the specific parameters and observations concerning the performed space-time cluster analysis. (DOCX 88 kb)

Additional file 2: Detail of spatio-temporal clusters identified in Girardot, 2012–2014. Description of data: Table containing the details of the space-time clusters identified (year, start-end dates, radius, observed cases, affected population and description of urban conditions, and overlap with spatial clusters). (DOCX 63 kb)
