# Supplementary material for: Space and space-time distributions of dengue in a hyper-endemic urban space: the case of Girardot, Colombia
Source: BMC Infect Dis. 2017 Jul 24;17:512. doi: 10.1186/s12879-017-2610-7 (PMC5525249; doi:10.1186/s12879-017-2610-7)
Supplement: Supplementary file 1 — Parameters for space-time cluster analysis (SaTScan V9.4.2). Description of data: Table containing the specific parameters and observations concerning the performed space-time cluster analysis. (DOCX 88 kb) [file 12879_2017_2610_MOESM1_ESM.docx]

Parameters for space-time cluster analysis (SaTScan V9.4.2)

| **Parameter** | | **Observations** |
| --- | --- | --- |
| Study period | 01/01/2012 – 31/12/2015 | Independent analyses were performed for each year and for the whole period.  Onset of symptoms date was used to define the study period |
| Spatial information | Coordinates (Latitude/Longitude) | Coordinates of block centroids were calculated using “inside polygon” option (ArcGIS 10.0) |
| Design | Type of Analysis:  Retrospective Space-Time  Probability Model:  Discrete Poisson  Scan for Areas with: High Rates  Time Aggregation: 20 Days |  |
| Spatial Window | Geometry of window: Circular  Maximum Spatial Cluster Size:  50 percent of population at risk |  |
| Temporal Window | Minimum Temporal Cluster Size: 1 Day  Maximum Temporal Cluster Size: 20 Days | Corresponds to vector-human-vector transmission cycle |
| Inference (Monte Carlo simulations) | P-Value Reporting: Default Combination  Number of Replications: 999 |  |
| Spatial Output | Report Hierarchical Clusters: Yes    Criteria for Reporting Secondary Clusters: No Geographical Overlap  Reported Clusters:  - Only clusters smaller than 50 percent of population at risk reported, or  - Only clusters smaller than 0.3 km | As explained in the software’s manual (Kulldorff, 2015), if a specific maximum cluster size is to be evaluated, it should be defined in the output parameters and not in the spatial window settings. This allows to have a proper statistical evaluation of all possible cluster sizes but only report the clusters with a maximum size defined by user. This is to avoid pre-selection bias.  A radius of 300 meters was selected as maximum size to report clusters, considering that maximum distances between blocks centroids are around 300 meters. In this way is assured that all blocks have at least 1 neighbor. |
