# Supplementary material for: Space and space-time distributions of dengue in a hyper-endemic urban space: the case of Girardot, Colombia
Source: BMC Infect Dis. 2017 Jul 24;17:512. doi: 10.1186/s12879-017-2610-7 (PMC5525249; doi:10.1186/s12879-017-2610-7)
Supplement: Supplementary file 2 — Detail of spatio-temporal clusters identified in Girardot, 2012–2014. Description of data: Table containing the details of the space-time clusters identified (year, start-end dates, radius, observed cases, affected population and description of urban conditions, and overlap with spatial clusters). (DOCX 63 kb) [file 12879_2017_2610_MOESM2_ESM.docx]

Detail of spatio-temporal clusters identified in Girardot, 2012-2014

| **Year** | **Time (Start-End date)** | **Space (Radius)** | **Observed cases** | **Affected population and description of urban conditions** | **Overlap with spatial clusters** |
| --- | --- | --- | --- | --- | --- |
| 2012 | 7-Mar to 26-Mar Rainy season | 98.9 meters | 5 | Most cases <15 years (2 cases > 15 years), 3/5 Male  Part of Santafe and Brisas del Bogotá neighbourhood. Formal, even and middle socioeconomic conditions | No |
| 2012 | 22-Nov to 11-Dec  End of rainy season | 228.8 meters | 6 | All cases <15 years, 3/6 Male  Part of Portachuelo neighbourhood. Heterogenic low-medium socioeconomic conditions, formal and informal residences, abandoned open spaces | Yes |
| 2012 | 12-Dec to 31-Dec  Dry season | 184.2 meters | 7 | Most cases <15 years (2 cases >15 years), 5/7 Male  Fragments of Conjunto El Refugio, San Fernando and Urbanización La Esmeralda. Heterogenic low to high socioeconomic conditions, informal, formal and second residences complex (high stratum). An abandoned and polluted canal divides Urbanización la Esmeralda from the rest of the cluster | Yes |
| 2013 | 1-Jan to 25-Jan  Dry season | 157.0 meters | 9 | Most cases <15 years (3 cases >15 years), 6/9 Male  Neighborhood El Diamante presents medium socioeconomic conditions throughout the cluster. Part of a large school lays inside the cluster. | No |
| 2013 | 1-Jan to 25-Jan  Dry season | 266.9 meters | 12 | 50% cases <15 years, 4/12 Male  Involves all of Urbanización Santa Paula, Villa Vanessa and a major part of Santa Isabel. Heterogenic middle to high socioeconomic conditions, including a second residence complex. | Yes |
| 2013 | 1-Jan to 25-Jan  Dry season | 150.0 meters | 5 | Most cases <15 years (1 case >15 years), 3/5 Male  Agua Blanca and Brisas de Guadalquivir are high-stratum second residence complex. Urbanización Villampis is a middle-high residential area. This cluster has overall good socioeconomic conditions. | Yes |
| 2013 | 15-Feb to 6-Mar  End dry season | 234.7 meters | 9 | Less than half of cases <15 years (5 cases >15 years), 4/9 Male  Part of La Esperanza and Hacienda Girardot, both share medium socioeconomic conditions. La Esperanza has a particular morphology with mainly pedestrian narrow streets to access the inner parts of the neighbourhood | Yes |
| 2013 | 6-May to 25-May Rainy season | 58.2 meters | 5 | Most cases <15 years (1 case >15 years) 2/5 Male  Involves a few blocks of El Triunfo neighbourhood. Medium socioeconomic conditions are present | No |
| 2014 | 26-Jan to 14-Feb  Dry season | 101.7 meters | 5 | Most cases <15 years (1 case >15 years), 3/5 Male  Part of Centenario neighbourhood, which presents medium socioeconomic conditions | No |
| 2014 | 22-Nov to 11-Dec  End of rainy season | 33.5 meters | 6 | 50% cases <15 years, 50% Male  This is the smallest and most northern cluster. Practically it covers 1 block of Ciudad Montes neighbourhood, where Mediumm-low conditions are present. | No |
